# Gut Virome of Tibetan Pigs Reveals the Diversity, Composition, and Distribution of Potential Novel Viruses/Variants

**DOI:** 10.1155/tbed/5191656

**Published:** 2025-11-19

**Authors:** Jiaheng Chen, Ga Gong, Shiyin Huang, Yue Chen, Shixing Yang, Quan Shen, Xiaochun Wang, Ping Wu, Yuwei Liu, Likai Ji, Wen Zhang

**Affiliations:** ^1^Department of Laboratory Medicine, School of Medicine, Jiangsu University, Zhenjiang, China; ^2^Animal Science College, Tibet Agriculture and Animal Husbandry University, Nyingchi, Tibet, China

**Keywords:** gut viral communities, metagenomics, phylogenetic analysis, qinghai-tibet plateau, tibetan pigs, viral diversity

## Abstract

As a local breed adapted to the extreme environment of the Tibetan Plateau, Tibetan pigs have not yet been systematically characterized in terms of their gut viral communities. In this study, we applied viral metagenomics to sequence fecal samples from 191 Tibetan pigs (including both healthy and diarrheal individuals) across four farms in Nyingchi, Tibet, aiming to reveal the diversity, composition, and distribution of gut viral communities in Tibetan pigs living at high altitudes. A total of nearly 120 million high-quality viral sequence reads were obtained, which were annotated into 16 viral families. The viral community was predominantly dominated by Microviridae, but its composition varied across different farms and health statuses. Phylogenetic analysis identified numerous virus sequences associated with pigs, including RNA viruses (such as Astroviridae (*n* = 7), Caliciviridae (*n* = 6), Picornaviridae (*n* = 15), etc.) and DNA viruses (such as Circoviridae (*n* = 3), Genomoviridae (*n* = 4), Smacoviridae (*n* = 41), Parvoviridae (*n* = 11), etc.). Notably, the study found multiple viral sequences exhibiting genetic differences from known strains, suggesting the potential presence of novel viruses or variants. For instance, a papain-like protease (PLP) insertion sequence, identified to have high sequence identity with *Torovirus* (ToV), was found in six *Enterovirus G* (EV-G) strains, indicating a cross-family genetic recombination event. This study systematically outlines the viral metagenomic profile of gut viral communities in Tibetan pigs at high altitudes, revealing their unique viral diversity and complex community structure. The results suggest that the gut viral community of Tibetan pigs consists of host-associated viruses, bacteriophages, and potentially viruses originating from the environment or diet, with its composition influenced by farming conditions and host health status. These findings provide an important data foundation for understanding the interactions between viruses, hosts, and the environment in unique ecological settings and offer new insights into the health management and virology research of Tibetan pigs.

## 1. Introduction

Tibetan pigs, a unique species native to the Tibetan Plateau in China, are primarily distributed in Tibet, Qinghai, and Ganzi Prefecture of Sichuan, and are typically found in high-altitude, cold, and hypoxic regions throughout the year [1, 2]. Their farming environment is drastically different from that of lowland pig breeds. The plateau climate is harsh, with low oxygen levels, consistently low temperatures, and intense ultraviolet radiation [3]. Moreover, due to the sparse vegetation and limited feed resources in these high-altitude regions, Tibetan pigs primarily rely on low-nutrient foods such as barley straw and high-altitude weeds. Their daily activity range can extend over several dozen square kilometers, and this high-intensity exercise results in muscle fiber density significantly higher than that of ordinary pigs [4]. Over thousands of years of natural selection and artificial breeding, Tibetan pigs have developed unique genetic traits and physiological mechanisms adapted to the extreme plateau environment, including resilience to coarse feed, strong disease resistance, and tender, nutritious meat [5–7]. Additionally, Tibetan pigs exhibit a significantly higher density of melanocytes in their skin compared to ordinary pigs, which provides effective protection against intense UV radiation [8]. Long-term adaptation to hypoxic conditions has also enhanced the functional capacity of their cardiovascular and respiratory systems, aiding in the maintenance of metabolic stability [9]. These traits not only reflect long-term adaptation to a unique ecological environment but may also influence the composition of their gut viral communities.

Diarrhea, one of the most common diseases in the pig industry, especially during the piglet stage, poses a serious threat to pig growth, immune function, and survival rates [10, 11]. Known viruses that cause diarrhea in pigs include porcine epidemic diarrhea virus (PEDV) [12], classical swine fever virus (CSFV) [13], porcine rotavirus (PoRV) [14], and porcine circovirus (PCV) [15], among others. These viruses are widespread in the general pig population, with high incidence rates and rapid transmission. For instance, PEDV outbreaks are more common during the cold season, leading to acute diarrhea, vomiting, dehydration, and even mortality rates as high as 100% in piglets [16, 17].

While studies have analyzed the imbalance and changes in the gut microbiota of diarrhea-affected Tibetan pigs [18], there is still a lack of systematic and in-depth research on the distribution, species composition, and specific effects of diarrhea-associated viruses on the host's health in Tibetan pigs. In addition to the common intestinal viral infections found in farming, Tibetan pigs may also be susceptible to various zoonotic viruses closely related to public health [19, 20]. Pigs are natural hosts for several viruses in viral ecology, playing a crucial role in interspecies transmission, where they can act as hosts and potentially transmit viruses to humans, posing a public health risk [21]. Therefore, a comprehensive analysis of the diversity of gut viruses in Tibetan pigs is important for understanding their health status and disease spectrum, while also providing a scientific basis for assessing the potential transmission risks of zoonotic diseases.

Although traditional virus detection methods, such as PCR and virus isolation offer advantages in specificity, they are limited in sensitivity. These methods can only identify known viruses and fail to comprehensively reflect the viral diversity within the host. The rise of next-generation sequencing (NGS) has provided a novel technical approach for viromics research [22, 23]. This method does not rely on preset detection targets and can acquire the entire genetic information of microorganisms in a sample, enabling not only the detection of known viruses but also the discovery of novel or variant strains. Additionally, it can be combined with phylogenetic analysis to reveal the evolutionary relationships of viruses [24].

To fill the gap in viral ecology research in extreme environments, this study focused on Tibetan pigs from four farms in Nyingchi, Tibet. A total of 191 fecal samples were collected, and 38 metagenomic libraries were constructed, covering both healthy and diarrheal individuals. Through metagenomic sequencing and bioinformatics analysis, we compared the composition and relative abundance differences of gut viral communities in Tibetan pigs across different farms and health statuses. A phylogenetic tree was constructed to analyze the genetic diversity of the major viral taxa, providing data support for the prevention and control of animal epidemics and public health safety.

## 2. Materials and Methods

### 2.1. Sample Collection

The objective of this study was to investigate the virome of 191 Tibetan pig fecal samples collected from four different regions (Xueba Village, Zengba Village Xinchang, Jiebucai Village, and Zengba Village Farming Cooperative) in Nyingchi, Tibet, in 2024. All Tibetan pigs were artificially raised, with ages not exceeding 12 months. The cohort included pigs at different developmental stages, with ages ranging from 0–40 days to 10–12 months old (Supporting Information 1: Table S1). Fecal samples were collected within 5 min after natural defecation using disposable gloves and placed in sterile containers. The samples were then transported on dry ice to the laboratory with the total time from collection to laboratory storage within 48 h. Prior to viral metagenomic analysis, all samples were homogenized and subjected to three freeze–thaw cycles. Then, 100 g of each sample was resuspended in 0.5 mL of Dulbecco's phosphate-buffered saline (DPBS), vigorously vortexed for 5 min, followed by centrifugation at 4°C, 15,000 *g* for 10 min. The supernatant was then transferred to 1.5 mL centrifuge tubes and stored at −80°C for nucleic acid extraction [25]. All samples were transported to the School of Medicine, Jiangsu University, where sample preparation was carried out in a biosafety level 2 laboratory.

### 2.2. Sample Preparation and Library Construction

Approximately 100 *μ*L of supernatant was first collected from each individual sample. Subsequently, based on the collection regions, clinical symptoms, and developmental stages of the pigs, these 191 samples were pooled into 38 pools. To ensure that each original sample contributed proportionally to the final pooled volume (~500 *μ*L per pool), the volume of supernatant taken from each sample was adjusted based on the number of samples in the pool: 100 *μ*L per sample for 5-sample pools, approximately 85 *μ*L per sample for 6-sample pools, and approximately 125 *μ*L per sample for 4-sample pools (Supporting Information 1: Table S1). The pooled samples were centrifuged at 13,000 *g* for 5 min at 4°C. The supernatant was then passed through a 0.45 *μ*m filter (Millipore) to remove eukaryotic cells and enrich viral particles [26]. Subsequently, DNase and RNase were used to digest unprotected nucleic acids at 37°C for 60 min [27]. Total nucleic acids (DNA and RNA) were extracted using the QiAamp Viral RNA Minikit (QIAGEN) according to the manufacturer's instructions. Reverse transcription was performed using SuperScript IV (Invitrogen) and 100 pmol random hexamer primers, converting the nucleic acids containing both DNA and RNA virus sequences into complementary DNA. Single-round DNA synthesis was then carried out using Klenow fragment polymerase (New England BioLabs) [28]. A total of 38 libraries were constructed using the Nextera XT DNA Sample Preparation Kit (Illumina), and sequencing was performed on the Illumina NovaSeq 6000 platform using 150 bp paired-end reads with dual barcoding [29].

### 2.3. Bioinformatics Analysis

The 150 bp paired-end reads generated from sequencing were paired using software provided by Illumina. The data were processed on an internal analysis pipeline running on a 32-node Linux cluster. First, the reference genome sequence of *Sus scrofa* (GenBank No. GCF_000003025.6) was downloaded from the NCBI database. Subsequently, we used Bowtie2 v2.5.0 to align and remove potential host sequences from the 38 libraries [30]. Primer sequences and low-quality reads were removed using Trim Galore v0.6.7 [31] (—phred33—length 100—stringency 3—paired), retaining only high-quality core sequence fragments. The reads were then assembled de novo using MEGAHIT v1.2.9 [32] (–min-contig-len 200), generating the longest contigs and singlets. The assembled contigs and raw reads were aligned against the NCBI viral protein database using DIAMOND v2.1.12 [33]. In addition to the default parameters, the alignment was performed with an E-value threshold of 1e − 5, and the options -k 25 and -f 100 were used to retain up to 25 candidate matches per query and to generate alignment outputs in DAA format, respectively. These DAA files were subsequently imported into MEGAN v7.1.1 [34] and converted into RMA format for viral community analysis. To further eliminate potential false-positive viral sequences, the filtered viral sequences were re-aligned against the internal nonredundant, nonviral protein (NVNR) database (downloaded in March 2025). All other parameters were kept at their default settings.

### 2.4. Viral Community Analysis

Statistical analyses were performed using MEGAN v7.1.1, R v4.5.1, and STAMP v2.1.3. The sequencing results from the 38 libraries were normalized, and species accumulation curves were plotted using the built-in rarefaction algorithm in MEGAN. Subsequent viral community statistics and visualization were conducted in R. The vegan package was employed for alpha- and beta-diversity analysis [35]. The ggplot2 package was used to generate all publication-quality figures, including boxplots, bar charts, and PCoA plots [36]. The UpSetR package was utilized to create the UpSet plot [37], and the bipartite network was visualized using the igraph package [38]. Differential abundance analysis between groups was performed using STAMP [39].

### 2.5. Viral Sequence Extension, Annotation, and Phylogenetic Analysis

Contigs with significant similarity to known viruses were selected based on BLASTx results. Contigs longer than 500 bp were further analyzed, using each contig as a reference for mapping raw data from its original barcode with the low sensitivity/Fastest parameter in Geneious Prime v.2024.0.5. Using Geneious Prime v.2024.0.5, we predicted specific open reading frames (ORFs) from complete or near-complete contigs that showed similarity to known viruses. ORFs were defined with a minimum size of 400 bp and an ATG initiation codon. These ORFs were validated by aligning them with viral proteins from the GenBank database. ORF annotation was performed using the Conserved Domain Database (CDD v3.21) (https://www.ncbi.nlm.nih.gov/Structure/cdd/wrpsb.cgi) [40–42]. Phylogenetic analysis was conducted based on the predicted protein sequences of viruses identified in this study and the most closely matching sequences obtained from blastx alignment (*E*-value cut-off < 10^−5^) against the GenBank database, along with representative viral sequences from related virus genera or species. MEGA v.11 [43] was used for MUSCLE alignment of the related protein and nucleotide sequences with default settings. The alignment was then trimmed using TrimAl v1.5.0 [44] (-automated1) to reduce noise, and phylogenetic trees were constructed using MrBayes v.3.2.7 [45]. Markov chain Monte Carlo (MCMC) parallel sampling was set up twice with the “prset aamodelpr = mixed” option for protein sequence-based phylogenetic analysis, and “lset nst = 6 rates = invgamma” for nucleotide sequence-based analysis. The runs were terminated when the split frequency standard deviation was less than 0.01, and the first 25% of the trees were discarded as burn-in. The phylogenetic trees were visualized and enhanced using ChiPlot [46] (https://www.chiplot.online/) and Adobe Illustrator 2025 v29.0. Host silhouettes on the phylogenetic trees were sourced from PhyloPic (https://www.phylopic.org/).

### 2.6. Quality Control

All experimental procedures were conducted following standard precautionary measures. Nucleic acid samples were dissolved in DEPC-treated water and RNase inhibitors were added. Double-distilled water (ddH_2_O) (Sangon Biotech) was prepared under the same conditions as blank controls for further processing. In addition, all pipette tips used during the experiment were equipped with aerosol filters, and all experimental materials that came into contact with nucleic acid samples, such as 1.5 mL centrifuge tubes and pipette tips, were RNase- and DNase-free. We implemented these procedures to prevent cross-sample contamination and nucleic acid degradation.

## 3. Results

### 3.1. Overall View of the Virome

As shown in Supporting Information 1: Table S1, all Tibetan pig fecal samples were collected from Nyingchi, Tibet, from four different farms: Xueba Village (Farm A), Zengba Village Xinchang (Farm B), Jiebucai Village (Farm C), and Zengba Village Farming Cooperative (Farm D). A total of 38 libraries were constructed, which generated 940,676,144 raw reads, averaging 24.75 million reads per library, with an average GC content of 39.7%.

Among these reads, 119,984,228 reads (12.75% of raw reads, *E*-value < 1e − 5) were matched to viral proteins using BLASTx based on protein sequence similarity. Species accumulation and rarefaction curves illustrated the species richness in the Tibetan pig fecal samples (Supporting Information 2: Figure S1A,B). We observed that the viral species in all sample pools eventually plateaued when the sequencing depth exceeded 10 million reads, indicating that the sequencing depth had sufficiently covered all viruses in the samples, and further sequencing would not reveal new viral species. The species accumulation curve reached a plateau when more than 80% of the total samples were included, and the slope of the curve was less than 0.05, indicating that the sampling effort was sufficient. This saturation suggests that the viral community in Tibetan pig feces was adequately represented, with approximately 80 distinct viral species potentially distributed across the 38 pooled samples.

**Figure 1:**
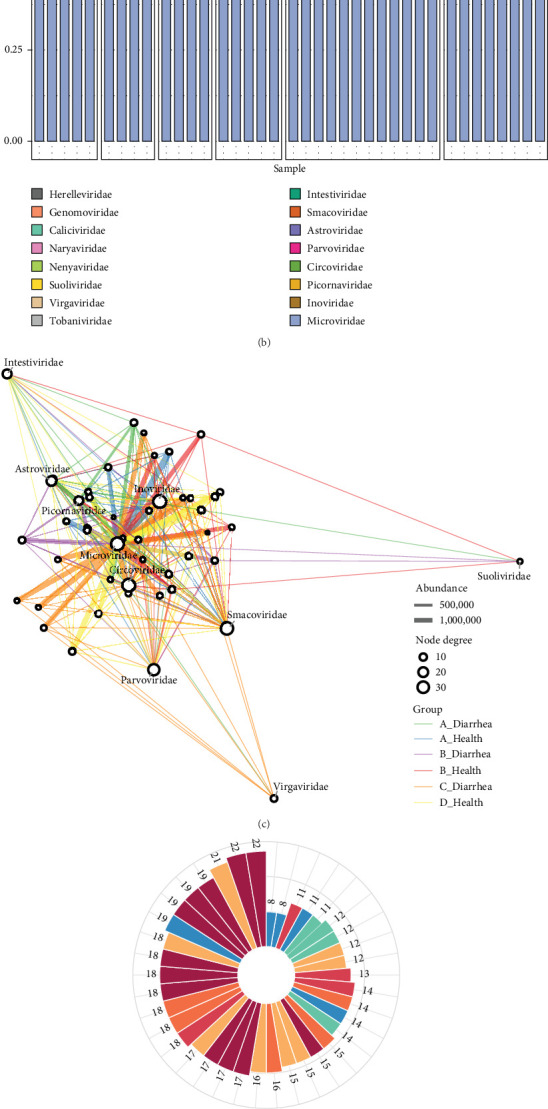
Virus classification at the family or species level. (A) Heatmap showing the read count for each viral family across all libraries from the four farms. The color blocks on the left represent different viral types, with the legend on the right. The row names on the right correspond to the viral family names. The data are presented on a logarithmic scale, with a base of log_10_. (B) Bar chart displaying the relative proportions and classifications of viral families, with the categories at the top representing the health and diarrhea conditions of Tibetan pigs from different farms. (C) Bipartite network graph depicting the relationship between different libraries, groups, and known viral families. The top 10 viral families with the highest connectivity were selected. Edge colors represent different groups, edge thickness indicates varying abundance, and node size reflects connectivity. (D) Rose diagram showing the number of viral species in each library. (E) Upset plot illustrating the number of viral species shared among the four farms and six groups. The horizontal bars on the left represent the number of viral species within each group. Lines connecting these points indicate the intersections between groups, with individual points corresponding to unique viral species within a group. Nodes connected by vertical lines represent shared viral species, and the bar chart shows the number of elements at the intersection points.

Based on the similarity to known viral sequences, the obtained contigs and singlets were classified into 16 viral families (Figure 1A). The results showed that Microviridae dominated across all libraries, including five RNA virus families (ssRNA) and 11 DNA virus families (three dsDNA and eight ssDNA). The samples were further divided into six subgroups based on the health and diarrhea conditions of the pigs from the four regions (A_Health, A_Diarrhea, B_Health, B_Diarrhea, C_Diarrhea, and D_Health). The stacked bar plot of abundance revealed that, in addition to the abundant Microviridae found in all libraries, Picornaviridae was significantly prevalent in the diarrheal pigs from Farms A and B. In Farms C and D, Microviridae, Inoviridae, and Circoviridae were predominantly observed (Figure 1B).

Furthermore, we constructed a bipartite network graph of the library-virus families and selected the top 10 viral families with the highest connectivity (Figure 1C). The results showed a complex interconnection of various viral families across different sample groups. Some viral families, such as Microviridae and Circoviridae, had high abundance and node degree, positioning them at the core of the network, reflecting their significant role in the viral community composition of Tibetan pig feces. In contrast, families, like Intestiviridae and Suoliviridae had lower connectivity, indicating a more limited distribution within the community.

Additionally, within the viral communities of the four farms, we identified 74 viral species at the species level and displayed their abundance distribution across the groups using a bubble plot (Supporting Information 2 Figure S1C). The Upset and polar coordinate plots revealed distinct viral community characteristics (Figure 1D,E). The results showed that nine viral species were shared across all four farms under different health and diarrhea conditions. Moreover, we found 14 unique viral species in the diarrheal pigs from Farm C, and the polar coordinate plot indicated that most libraries from Farm C had higher numbers of viral species than those from other farms. This suggests that the viral community in Farm C may be more complex and unique.

### 3.2. Differences and Distribution of Viruses in the Gut Virome of Tibetan Pigs

**Figure 2:**
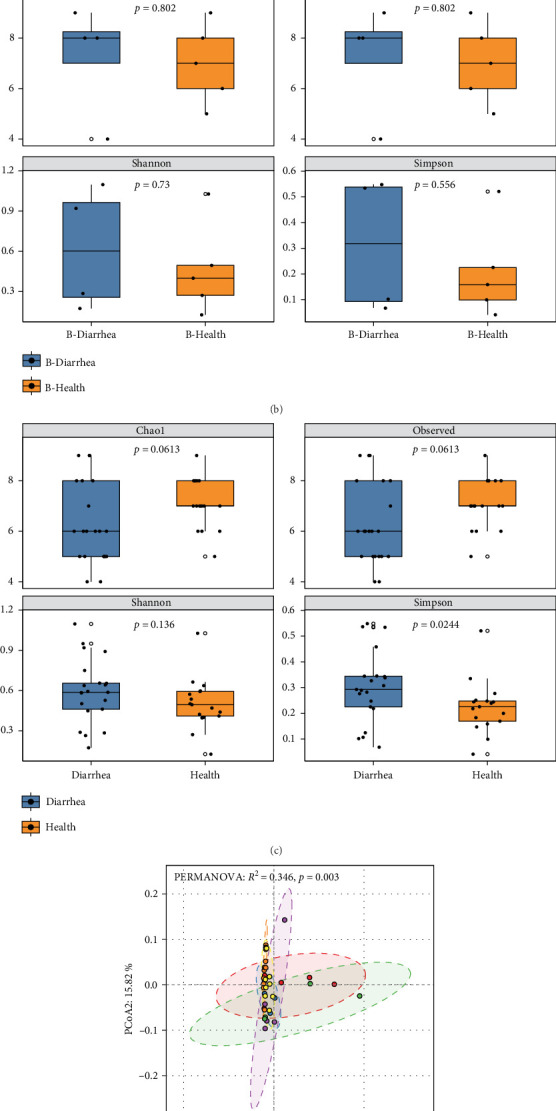
Viral community diversity in Tibetan pigs from Farm A, Farm B, and All farms under healthy and diarrhea conditions. (A) Boxplot showing the *α*-diversity comparison at the family level between healthy and diarrhea Tibetan pigs from Farm A. The *p*-value indicates whether there is a significant difference in the diversity index within the groups. (B) Boxplot showing the *α*-diversity comparison at the family level between healthy and diarrhea Tibetan pigs from Farm B. The *p*-value indicates whether there is a significant difference in the diversity index within the groups. (C) Boxplot showing the *α*-diversity comparison at the family level between all healthy and diarrhea Tibetan pigs. The *p*-value indicates whether there is a significant difference in the diversity index within the groups. (D) PCoA analysis comparing the viral *β*-diversity between healthy and diarrhea Tibetan pigs from the four farms. (E) Stamp analysis showing the viral family differences between healthy and diarrhea Tibetan pigs from the four farms.

To further investigate the differences and distribution characteristics of gut viral communities in Tibetan pigs across different farms and health statuses (healthy vs., diarrheal), we performed a comparative analysis of viral abundance and composition. The *α*-diversity results showed that, in Farms A and B, no significant differences were observed between healthy and diarrheal pigs in terms of Chao1, Observed, Shannon, and Simpson indices (*p* > 0.05) (Figure 2A, B). When all farms were combined for comparison, a significant difference was found only in the Simpson index (*p* < 0.05) (Figure 2C). Further *β*-diversity analysis revealed significant differences in the viral community structure between healthy and diarrheal pigs across the four farms (*p* < 0.01) (Figure 2D). Additionally, the extended error bar analysis based on STAMP showed significant abundance differences in Microviridae, Virgaviridae, and Inoviridae after combining all farm samples (Figure 2E). These results suggest that although no significant differences were observed in some diversity indices within individual farms, there were still significant differences in the viral community structure and the distribution of specific viral families between healthy and diarrheal Tibetan pigs at the overall level.

### 3.3. Novel RNA Viruses Detected in the Gut Virome of Tibetan Pigs

In this study, we identified a total of 48 different RNA virus genomes from Tibetan pig feces, including Astroviridae (*n* = 7), Caliciviridae (*n* = 6), Totiviridae (*n* = 1), Picornaviridae (*n* = 15), Tobanviridae (*n* = 1), Picobirnaviridae (*n* = 6), Dicistroviridae (*n* = 1), and unclassified RNA viruses (*n* = 11).

**Figure 3:**
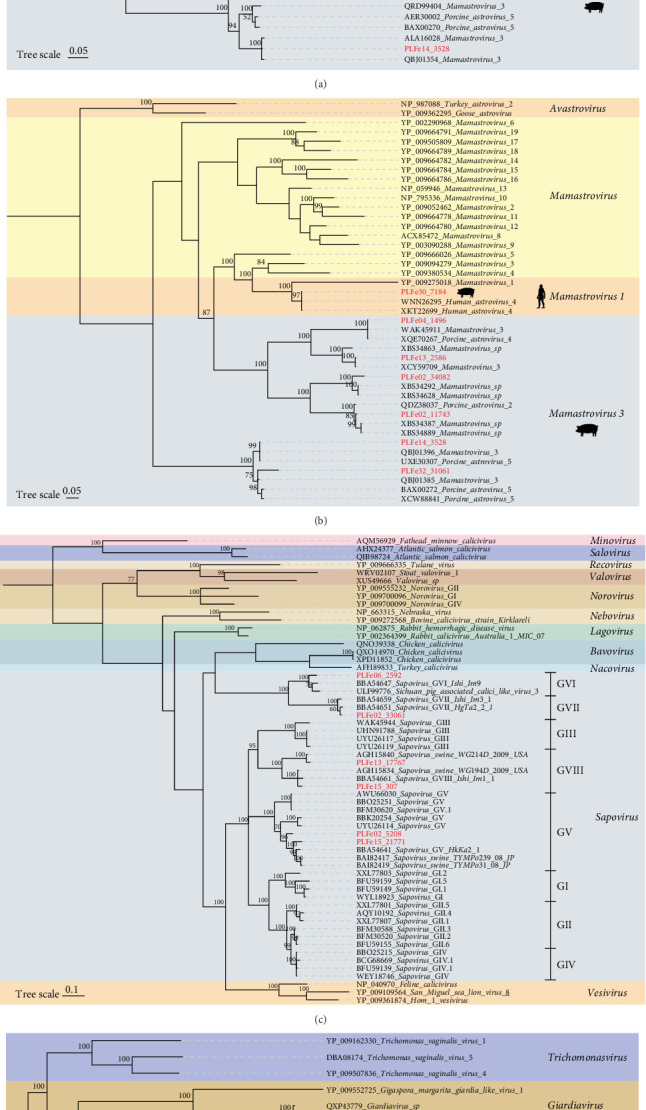
Phylogenetic analysis of Astroviridae, Caliciviridae, and Totiviridae Families in Tibetan pigs. (A) Phylogenetic tree of the Astroviridae family constructed based on the RdRp protein amino acid sequences. (B) Phylogenetic tree of the Astroviridae family constructed based on the Cap protein amino acid sequences. (C) Phylogenetic tree of the Caliciviridae family constructed based on the RdRp protein amino acid sequences. The purple color blocks represent different genetic groups of Sapovirus. (D) Phylogenetic tree of the Totiviridae family constructed based on the RdRp protein amino acid sequences. The color blocks on all trees represent different viral genera within each viral family, with the red highlighted fonts indicating newly discovered viruses in this study. Scale bar indicates the number of amino acid substitutions per site.

In this study, we characterized seven Astroviruses from 38 libraries, all of which contained complete genomes. A phylogenetic tree based on RNA-dependent RNA polymerase (RdRp) was constructed (Figure 3A), revealing that six of the strains showed the highest amino acid identity (92.75%–99.59%) with *Mamastrovirus* 3 virus sequences. Notably, PLFe30_7184 clustered with genus *Mamastrovirus* 1, showing 100% amino acid identity with human astrovirus (GenBank No. XPU89992). Additionally, we constructed a phylogenetic tree based on the capsid protein (Figure 3B), which yielded similar results to the RdRp analysis. PLFe30_7184 exhibited 99.73% amino acid identity with human astrovirus (GenBank No. XKT22699), while the remaining six strains showed 73.58%–100% identity with *Mamastrovirus* 3 virus sequences.

In this study, we assembled six related viral sequences, three of which had complete genomes (PLFe02_5208, PLFe02_33061, and PLFe13_17767). All sequences contained complete RdRp structures, and a phylogenetic tree based on this protein was constructed (Figure 3C). The results showed that the six viral sequences clustered with known sapovirus sequences in the public database, with amino acid identity ranging from 81.14% to 96.16%, and were spread across the known sapovirus genogroups GV, GVI, GVII, and GVIII. We also assembled an incomplete genome of a totivirus (PLFe13_18681) from Tibetan pig feces, with a sequence length of 4070 nt and a 3′ end missing. A phylogenetic tree based on RdRp was constructed (Figure 3D), and the sequence was found to form a separate small branch with a totivirus from pig feces (GenBank No. UHS72459), showing 95.34% amino acid identity.

**Figure 4:**
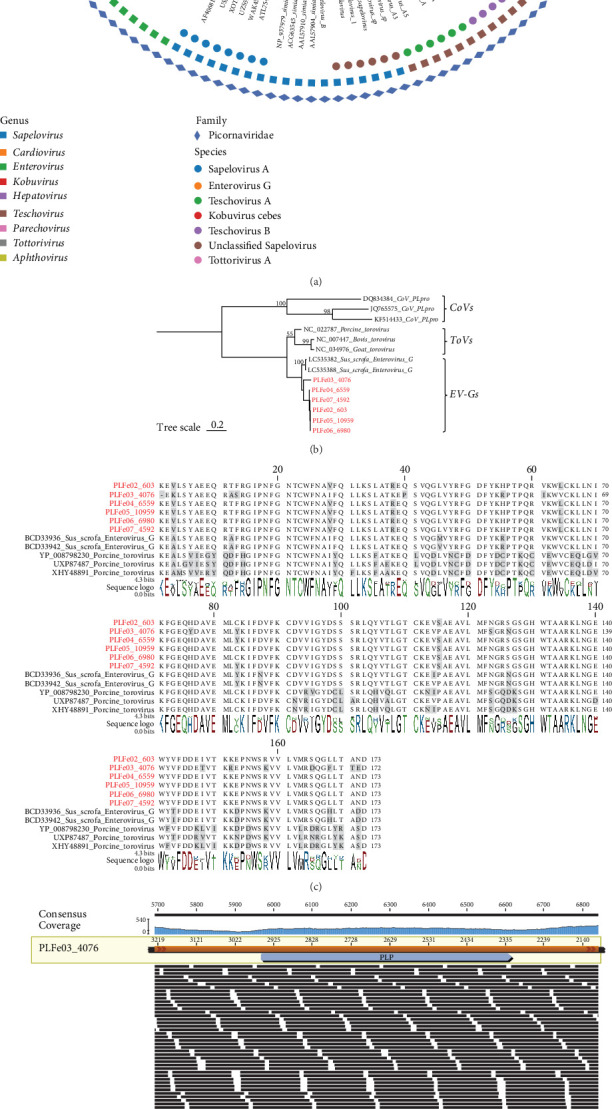
Phylogenetic analysis of Picornaviridae and Tobanviridae Families in Tibetan pigs. (A) Phylogenetic tree of the Picornaviridae family constructed based on the RdRp protein amino acid sequences. Rhombus, square, and circle legends represent viral families, genera, and species, respectively, with specific names provided in the upper left corner. (B) Phylogenetic tree constructed based on the nucleotide sequence of the inserted fragment (PLP). Red highlighted fonts indicate sequences containing the inserted fragment. (C) Multiple sequence alignment of the inserted fragment amino acid sequences with known pig-related enterovirus and pig-related torovirus sequences containing similar insert fragments. (D) This shows the read mapping visualization results of a representative Enterovirus G strain (e.g., PLFe03_4076) using Geneious Prime software. The PLP insertion fragment is represented in blue, and the black bars correspond to raw reads. These raw reads cover the exact junction region between the viral backbone and the insertion fragment. (E) Phylogenetic tree of the Tobanviridae family constructed based on the RdRp protein amino acid sequences. Scale bar indicates the number of amino acid or nucleotide substitutions per site.

We obtained 15 newly detected picornaviruses strains from 38 libraries, 14 of which had complete viral genomes with nucleotide lengths ranging from 6930 to 8831 nt. A phylogenetic tree based on RdRp was constructed (Figure 4A), and the results showed that these 15 picornaviruses belonged to five different genera: *Enterovirus*, *Sapelovirus*, *Teschovirus*, *Tottorivirus*, and *Kobuvirus*, with amino acid sequence identities ranging from 86.6% to 99.72%. Surprisingly, we discovered that six strains contained a potential insertion of approximately 639 nt in length. Upon NCBI blastx analysis, we identified this inserted fragment as a papain-like protease (PLP) [47]. We then constructed a phylogenetic tree for this insertion sequence at the nucleotide level (Figure 4B) and performed multiple sequence alignments at the amino acid level with two Japanese-derived enterovirus recombinants and representative torovirus (ToV) sequences (Figure 4C), showing 82.7% sequence identity. Phylogenetic analysis revealed that PLPs from *Enterovirus G* (EV-G) and ToV formed an evolutionary branch, suggesting a possible cross-family recombination event. To confirm this was a true biological recombination and not an assembly artifact, we mapped the raw sequencing reads back to the assembled genomes of all six EV-G strains. Representative read mapping for one strain clearly shows multiple reads spanning both insertion junctions (Figure 4D), and identical validation was obtained for the remaining five strains (Supporting Information 3: Figure S2), collectively providing robust evidence for this cross-family recombination event.

Furthermore, we assembled a complete genome of a Tobanviridae family sequence from the 38 libraries. We constructed a phylogenetic tree based on RdRp (Figure 4E), which showed that this virus was closely related to the ToV genus, formerly classified as a coronavirus, with 99.1% amino acid identity to a pig-derived ToV sequence from Guangxi (GenBank No. QYF49650).

**Figure 5:**
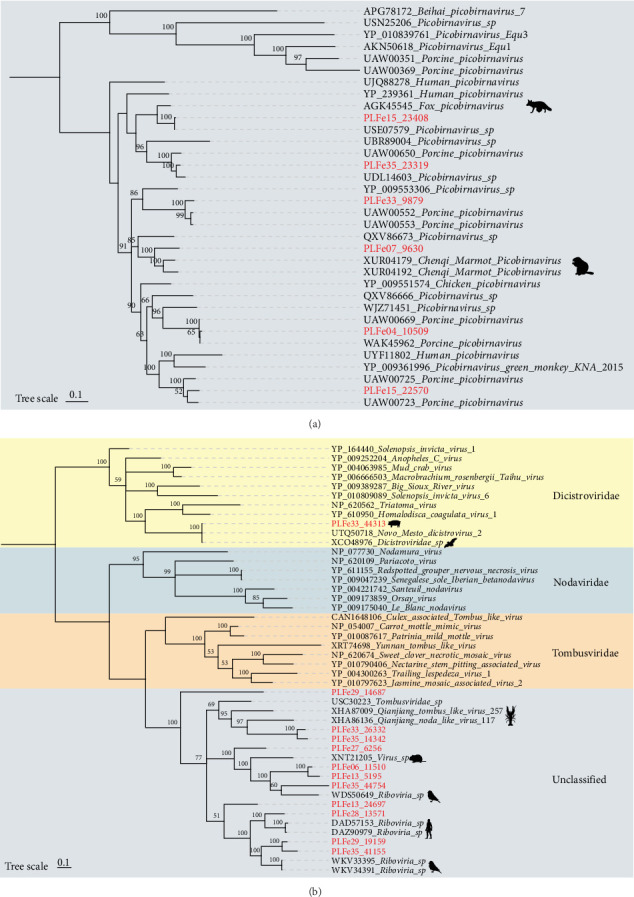
Phylogenetic analysis of Picobirnaviridae, Dicistroviridae, and unclassified RNA viruses in Tibetan pigs. (A) Phylogenetic tree of the Picobirnaviridae family constructed based on the RdRp protein amino acid sequences. (B) Phylogenetic tree of the Dicistroviridae family and unclassified RNA viruses constructed based on the RdRp protein amino acid sequences. Red highlighted labels indicate newly discovered viruses in this study. Color blocks represent different taxonomic groups. Scale bar indicates the number of amino acid substitutions per site.

In this study, we identified six picobirnavirus fragments, with amino acid identities to other known picobirnaviruses ranging from 76.13% to 99.23%. A phylogenetic tree based on RdRp was constructed (Figure 5A), showing that four of the picobirnaviruses clustered with picobirnaviruses identified from pig feces, while the remaining two strains, PLFe15_23408, clustered with picobirnaviruses from pig feces (GenBank No. USE07579) and fox feces (GenBank No. AGK45545); PLFe07_9630 clustered with two marmot-derived picobirnaviruses (GenBank No. XUR04179 and GenBank No. XUR04192), suggesting a possible close relationship between different vertebrates.

Additionally, we identified virus genomes related to Dicistroviridae, Nodaviridae, and Tombusviridae families in Tibetan pig feces, with RdRp proteins showing amino acid identities ranging from 34.39% to 99.37%. Phylogenetic trees constructed based on RdRp (Figure 5B) showed that most of these strains could not be classified into known genera of these families (classified as unclassified Riboviria). PLFe33_44313 formed a branch with known plant-derived dicistroviruses (GenBank No. UTQ50718) and bat-derived dicistroviruses (GenBank No. XCO48976), and exhibited an amino acid identity of 99.37% with both strains from these two sources.

### 3.4. Novel DNA Viruses Detected in the Gut Virome of Tibetan Pigs

In addition, we identified 61 DNA virus genomes from five viral families, including Circoviridae (*n* = 3), Genomoviridae (*n* = 4), Smacoviridae (*n* = 41), Parvoviridae (*n* = 11), and Anelloviridae (*n* = 2).

**Figure 6:**
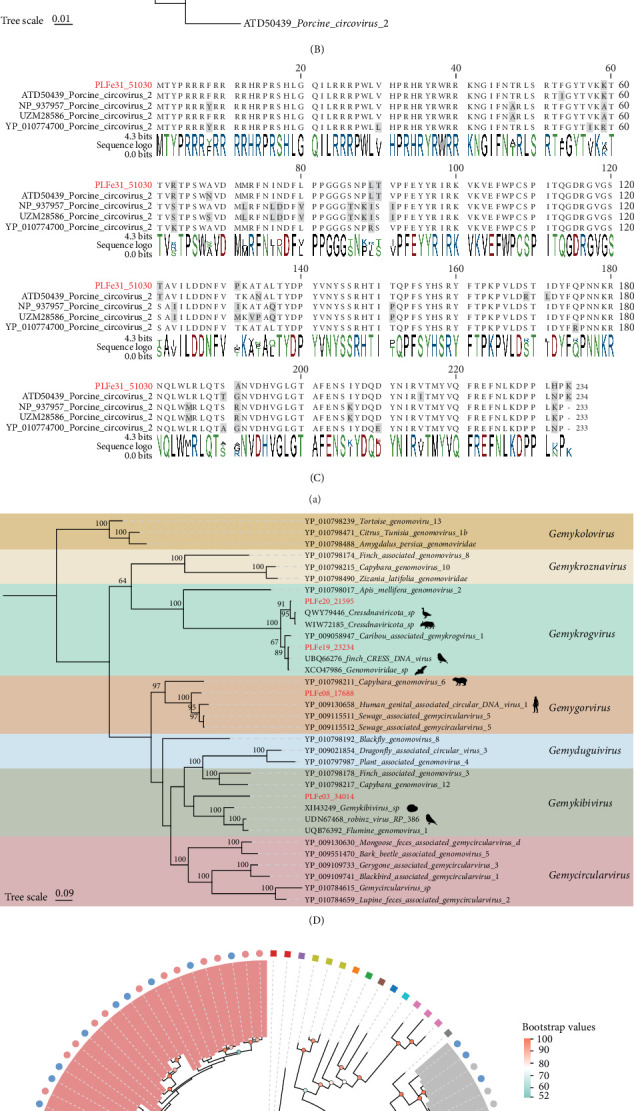
Phylogenetic analysis of CRESS DNA viruses in Tibetan pigs. (A) Phylogenetic tree of the Circoviridae family constructed based on the Rep protein amino acid sequences. (B) Phylogenetic tree of PCV2 virus constructed based on the Cap protein amino acid sequences. (C) Multiple sequence alignment of Cap protein amino acid sequences with known PCV2 virus sequences. (D) Phylogenetic tree of the Genomoviridae family constructed based on the Rep protein amino acid sequences. (E) Phylogenetic tree of the Smacoviridae family constructed based on the Rep protein amino acid sequences. Square and circle icons represent different taxonomic groups. Different color blocks also represent different taxonomic groups. Red highlighted labels indicate newly discovered viruses in this study. Scale bar indicates the number of amino acid substitutions per site.

In this study, we identified a total of 48 full genomes of cyclic replication-encoding single-stranded (CRESS) DNA viruses from 38 libraries. Among these, we assembled three circoviruses, and a phylogenetic tree based on the Rep protein was constructed (Figure 6A). The results showed that PLFe18_10456 and PLFe35_17506 did not cluster with known members of the *Cyclovirus* and *Circovirus* genera, formed a separate branch with porcine-related viruses showing high sequence identity, which classifies them as unclassified Circoviridae. In contrast, PLFe31_51030 clustered with the known PCV2 from the public database, exhibiting 100% amino acid identity with PCV2 (GenBank No. ATD50438). Subsequently, based on the virus strains from the PCV2 branch, we constructed a phylogenetic tree using the Cap protein from the full-genome sequence in the public NCBI database (Figure 6B). As with the previous results, PLFe31_51030 showed 95.7% amino acid identity with PCV2 (GenBank No. ATD50439). Multisequence alignment of Cap proteins from five strains showed a high degree of consistency, with an identity of 91.3% (Figure 6C). Therefore, we hypothesize that PLFe31_51030 likely belongs to a variant strain of PCV2, with potential local transmission and evolution within the pig population. We also assembled four full genomes of genomoviruses and constructed a phylogenetic tree based on the Rep protein (Figure 6D). These sequences exhibited 57.65%–99.51% amino acid identity with known genomoviruses, and phylogenetic analysis revealed that these four genomoviruses belonged to the genera *Gemykrogvirus*, *Gemygorvirus*, and *Gemykibivirus*. Interestingly, the viruses clustering with these four genomoviruses came from hosts other than pigs, including humans, birds, aquatic animals, other mammals, and even environmental samples, further supporting the wide cross-species host range of Genomoviridae. Additionally, we assembled 41 full genomes of smacoviruses, and a phylogenetic tree based on the Rep protein was constructed (Figure 6E). The majority of the smacoviruses in this study belonged to the genus *Porprismacovirus*, and all smacoviruses clustered with known pig-derived strains, with amino acid identities ranging from 67.36% to 99.26%.

**Figure 7:**
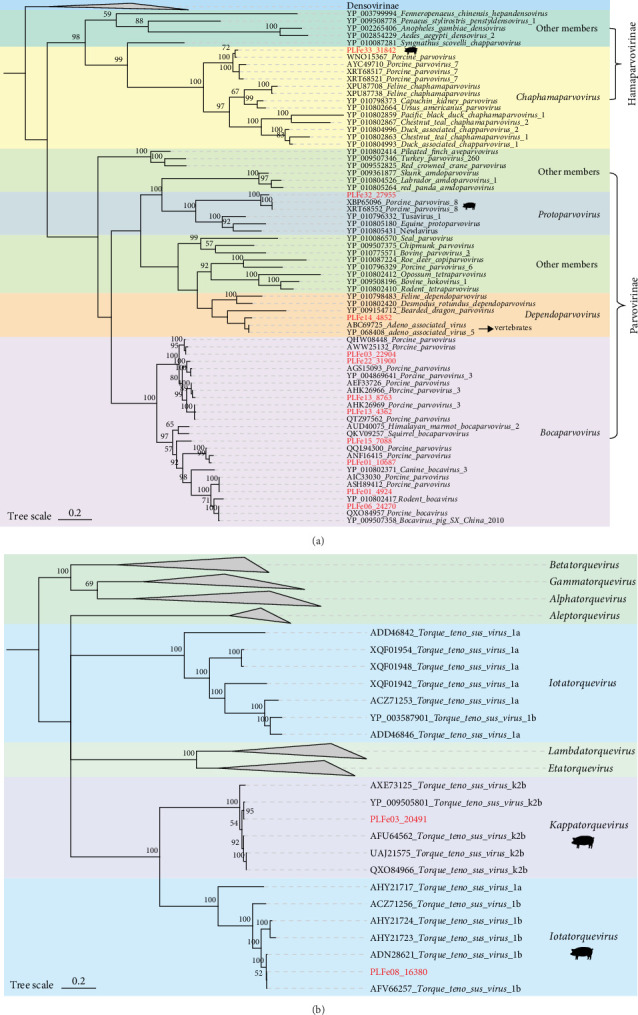
Phylogenetic analysis of Parvoviridae and Anelloviridae in Tibetan pigs. (A) Phylogenetic tree of the Parvoviridae family constructed based on the NS1 protein amino acid sequences. (B) Phylogenetic tree of the Anelloviridae family constructed based on the ORF1 protein amino acid sequences. Red highlighted labels indicate newly discovered viruses in this study. Different color blocks represent different taxonomic groups. Scale bar indicates the number of amino acid substitutions per site.

In this study, we assembled 11 full genomes of parvoviruses. A phylogenetic tree based on the NS1 protein was constructed (Figure 7A). We found that eight of the parvoviruses formed a large branch with known strains from the genus *Bocaparvovirus*, with amino acid identity ranging from 54.6% to 100%. Among the remaining three parvoviruses, PLFe33_31842 showed 95.68% amino acid identity with a porcine parvovirus (PPV) from the genus *Chaphamaparvovirus* (GenBank No. WNO15367); PLFe32_27955 showed 86.36% amino acid identity with a PPV from the genus *Protoparvovirus* (GenBank No. XBP65096); and PLFe14_4852 showed 84.26% amino acid identity with an adeno-associated virus (AAV) from the genus *Dependoparvovirus* (GenBank No. ABC69725). We also assembled two fragments of anelloviruses encoding only ORF1 and constructed a phylogenetic tree based on the ORF1 protein (Figure 7B). The results revealed that the two anelloviruses belonged to two different genera. PLFe03_20491 showed 99.08% amino acid identity with the reference strain of torque teno sus virus K2b (GenBank No. YP_009505801), and both clustered with *Kappatorquevirus* strains from pigs. PLFe08_16380 showed 99.83% amino acid identity with torque teno sus virus 1b (GenBank No. AFV66257) and clustered with *Iotatorquevirus* strains from pigs.

## 4. Discussion

Based on metagenomic data from Tibetan pig feces from the Linzhi region of Tibet, this study systematically analyzes the composition, distribution differences, and novel viruses in the gut virome. Compared to previous studies mainly focused on intensively farmed pig populations, the subjects of this study are local pig breeds that have been under semi-pastoral management for an extended period and live in a plateau ecological environment. This provides additional key evidence on the gut virome of pigs in different ecological settings. The sequencing depth was sufficient, and the community coverage was comprehensive, ensuring the reliability of the virome and phylogenetic analyses. Moreover, multiple novel viruses were identified, along with viral sequences potentially related to cross-species transmission, offering a new perspective on the role of Tibetan pigs as potential viral hosts and contributing to the understanding of viral diversity.

Microviridae phages were the dominant viral family across all samples, we considered whether this dominance could be an artifact of technical bias in our metagenomic workflow. However, we deem this unlikely for several reasons. The use of random hexamer primers during reverse transcription and the single-round DNA synthesis with Klenow fragment are designed to minimize sequence-specific amplification biases [48, 49]. More importantly, the preponderance of Microviridae is a well-documented and common feature in the fecal viromes of various mammals, as reported in numerous independent studies [50]. This alignment with their established role as key regulators of gut bacterial communities and intestinal homeostasis [51, 52]. Their prevalence may be linked to the unique gut microbiota of high-altitude-adapted Tibetan pigs [3, 5], which provides abundant bacterial hosts, as well as their semi-pastoral grazing practices that likely facilitate continuous environmental phage exposure [53]. This dominance was further underscored in diarrheal pigs, supported by a significant reduction in viral *α*-diversity (Simpson's index), indicating a virome dominated by fewer taxa, primarily Microviridae. In the diarrheal pig populations of Farms A and B, the relative abundance of *Picornaviridae* was higher, and this virus group has already been proven to be closely associated with porcine gastrointestinal diseases [54]. On the other hand, viral diversity and load were overall higher in diarrheal pigs, especially in the diarrheal pigs of Farm C, where the highest number of viral species was detected. This suggests that factors such as farming scale, density, and sanitary conditions may significantly influence the composition and spread of the virome [55].

Regarding RNA viruses, astroviruses consist of three overlapping ORFs, with ORF1a encoding a nonstructural polyprotein 1A, ORF1b encoding a nonstructural protein 1AB, primarily containing RdRp, and ORF2 encoding the viral capsid protein [56]. Astroviruses are often one of the leading causes of infantile diarrhea, and for pigs, these viruses are typically endemic, with fatal outcomes [57]. We identified seven strains of astroviruses, six of which were identified as being related to the porcine *Mamastrovirus* 3. The astrovirus strain PLFe30_7184 exhibits nearly complete identity to human strains, with 100% identity in the RdRp region and 99.73% in the capsid region. Laboratory contamination was ruled out, as no human samples or human astrovirus work are conducted in our facility. Therefore, its detection suggests potential cross-species transmission. However, the presence of viral DNA does not confirm active infection and may represent passive carriage. To assess its spillover potential, future studies should include conducting serological surveys in local pig and human populations to detect specific antibodies, attempting to isolate the virus using porcine cell lines, and performing experimental infection studies to evaluate pathogenicity. Caliciviruses can cause severe gastrointestinal symptoms in humans, and pigs serve as the primary host for sapoviruses, exhibiting symptoms like diarrhea and vomiting upon infection [58]. The six identified sapoviruses were classified into four genogroups (GV, GVI, GVII, and GVIII), consistent with previous reports [59].

Picornaviruses have a wide range of hosts and are primarily transmitted via the fecal-oral route, causing a variety of gastrointestinal diseases in mammals [60]. Among the 15 picornaviruses identified, we found six EV-G strains carrying an insertion sequence encoding a PLP that shared high amino acid identity (82.7%) with that of ToV, suggesting a horizontal gene transfer or recombination event across virus families. Similar phenomena have been reported in Japanese porcine EV-G viruses, which may increase viral genetic diversity and enhance environmental adaptability and pathogenic potential [61]. In addition, this study detected viruses, such as tobanvirus, picobirnavirus, and dicistrovirus. Tobanviridae have a wide host range and primarily infect vertebrates, causing a variety of diseases, including diarrhea and respiratory diseases [62]. One tobanvirus strain identified as highly similar to the Guangxi pig tobanvirus (amino acid identity of 99.1%), further supporting the widespread distribution of ToV in Chinese pig populations [63]. However, de novo validation revealed that the inserted fragment in picornaviruses did not originate from the ToV strain identified in this study. Moreover, Picobirnaviruses are small, nonenveloped viruses with a genome consisting of two segments, with the larger segment encoding the capsid protein and the smaller segment encoding RdRp. These viruses can infect vertebrates [64]. some picobirnaviruses sequence clustered in the phylogenetic tree with strains from wild animals, including foxes, marmots, and bats, suggesting that Tibetan pigs may be exposed to these viruses through shared environments or food. Moreover, In this study, a totivirus and a dicistrovirus were identified from the feces of Tibetan pigs. Totiviruses are typically known to infect fungi or protozoa [65], whereas dicistroviruses mainly infect insects and other arthropods [66]. The presence of these viruses suggests that they most likely originated from symbiotic or pathogenic microorganisms, or from dietary components rather than directly infecting pig cells. Specifically, PLFe33_44313 clustered closely with plant-derived (GenBank No. UTQ50718) and bat-derived (GenBank No. XCO48976) dicistroviruses, sharing 99.37% amino acid identity with both strains, while PLFe13_18681 exhibited 95.34% amino acid identity with a totivirus previously detected in pig feces (GenBank No. UHS72459). These viruses may have originated from infected insects or been introduced through the pigs' diet. Nevertheless, whether these viruses are capable of replication within Tibetan pigs remains to be further investigated [67, 68].

Regarding DNA viruses, CRESS DNA viruses have an extremely wide host range, capable of infecting nearly all eukaryotes [69]. PCV2 is a major pathogen causing PCV-associated diseases (PCVAD) globally, which lead to reproductive disorders, emaciation, respiratory distress, and other systemic diseases, causing significant economic losses [70]. In this study, we identified a circovirus genome (PLFe31_51030) whose Rep and Cap proteins exhibited high amino acid identity with known PCV2 strains (100% and 95.7%, respectively), suggesting local circulation and potential mutation of PCV2 in Tibetan pigs. The conservation of the Rep protein, essential for viral replication, indicates a stable replication mechanism [71]. In contrast, the amino acid substitutions in the major antigenic Cap protein could potentially alter viral antigenicity [72]. This divergence is significant, as the effectiveness of PCV2 vaccines, which have successfully reduced disease prevalence, can be limited by the emergence of such novel antigenic variants [73]. Consequently, serological studies are warranted to evaluate the cross-protective efficacy of existing vaccines against this Tibetan pig-derived strain. Notably, this strain was found in a healthy Tibetan pig from Farm D (Supporting Information 1: Table S1), suggesting that the strain might be in a latent infection stage. If the population remains healthy for an extended period, this strain could represent a low pathogenic or highly adaptive variant [74]. These findings could serve as a valuable reference for evaluating existing vaccines and developing new ones. Genomoviruses are a class of widely distributed single-stranded DNA viruses with a host range that includes fungi, plants, insects, and mammals, particularly in their intestinal environments [75]. Based on the phylogenetic tree, the Rep protein sequences of the four genomoviruses we identified showed high amino acid identity with reference strains from humans, birds, aquatic animals, and environmental samples, which further supports the virus's broad host range and potential for cross-species transmission. Smacoviruses, although their exact host is not yet defined, are commonly found in the intestinal metagenomes of various vertebrates, suggesting a potential association with intestinal microorganisms or host symbiosis. Most of the smacoviruses identified in this study belonged to the genus *Porprismacovirus*, indicating that this virus is relatively common in pig populations and exhibits some host specificity [76]. Parvoviridae is a common family of single-stranded DNA viruses, prevalent in vertebrates like pigs. PPV is mainly associated with reproductive disorders in pigs, while porcine bocavirus (PBoV) is frequently observed in coinfections [77, 78]. In addition to the commonly detected bocaviruses, we also identified members of genus *Chaphamaparvovirus*, *Protoparvovirus*, and *Dependoparvovirus*. One strain of genus *Dependoparvovirus* was identified as being closely related to AAV, and previous research has shown that AAV replication typically requires a helper virus. However, the ecological role and potential function of AAV in the Tibetan pig gut remain unclear and warrant further investigation [79]. Additionally, we identified two anelloviruses that were closely related to porcine TTV within the Anellovirus family. Previous studies have shown that TTV is widespread in pig populations and often co-infects with viruses, like PCV2, potentially exacerbating clinical symptoms through immune modulation [80]. However, since the PCV2 and anellovirus strains identified in this study were from different farms, direct inferences regarding their interaction are not yet possible, and further investigation in larger-scale studies is needed to assess potential associations.

However, this study has some limitations. First, all samples were collected from one geographic region (Nyingchi) in Tibet. While Nyingchi is a representative and major hub for Tibetan pig husbandry, the gut virome of pigs in other parts of the plateau may differ due to variations in altitude, climate, and management practices. Second, the sample size of Tibetan pig feces was relatively small, particularly with regard to farm distribution. Only Farms A and B contained both healthy and diarrheic individuals, while Farm C only had diarrheic pigs and Farm D only had healthy pigs (Supporting Information 1: Table S1). This sample distribution limits the comprehensiveness and robustness of the viral community comparison, potentially introducing bias in the interpretation of some results. Third, although our sequencing depth was sufficient to capture the dominant viral community, viruses present at low abundance may remain undetected [81, 82]. Furthermore, biases can be introduced during nucleic acid extraction and amplification, which may affect the representation of certain virus types in the final dataset [83]. Additionally, since the samples were collected solely from Tibetan pigs in high-altitude regions of Tibet, the unique ecological environment, climatic conditions, and traditional farming practices may have influenced the composition of the gut virome. Therefore, the applicability of these findings to other regions and farming systems should be considered with caution. Moreover, our viral enrichment workflow employed filtration through a 0.45 µm membrane, which is a common step for removing host cells and bacteria [84]. However, this approach may simultaneously exclude large-sized giant viruses (e.g., those belonging to the families Mimiviridae and Pandoraviridae), as their particle sizes sometimes exceed the filter pore diameter [85]. Consequently, the present study may underestimate the presence and diversity of giant DNA viruses in the gut of Tibetan pigs. Future studies that adopt filtration-free or size-fractionated enrichment strategies will help obtain a more comprehensive viral community profile [86, 87].

Overall, this study reveals the complexity and regional variation of the gut virome in Tibetan pigs in high-altitude environments. However, due to the limitations of sample size and other factors, further validation and expansion of these findings in broader, multilayered studies are necessary.

## 5. Conclusion

This study systematically analyzed the composition and diversity of the gut virome in Tibetan pigs from high-altitude regions of Tibet. The results revealed that the virome includes not only common animal-associated viruses but also potential novel or low-abundance viruses. Additionally, there were observable differences between regions and health statuses, suggesting that both environmental and host factors play a role in shaping the unique virome characteristics of Tibetan pigs. This research provides valuable data on the distribution patterns of gut viruses under high-altitude ecological conditions, filling a gap in the study of viral ecology in extreme climates. Furthermore, it offers a solid scientific foundation for pig farming management and disease prevention.

## Data Availability

The raw sequence data generated in this study have been deposited in the Genome Sequence Archive (GSA) of the National Genomics Data Center (NGDC), Beijing Institute of Genomics, Chinese Academy of Sciences/China National Center for Bioinformation (GSA: CRA028856; https://ngdc.cncb.ac.cn/gsa) [88]. Additional data have been deposited in GenBase at NGDC under accession numbers C_AA113742.1–C_AA113791.1 and C_AA113813.1–C_AA113871.1, and are publicly accessible at https://ngdc.cncb.ac.cn/genbase [89, 90].

## Supporting Information

Supporting Information 1 Table S1. Information of Tibetan pigs and libraries included in the present study.

Supporting Information 2 Figure S1. (A) Rarefaction curves of species assignments generated in MEGAN v7.1.1 after log transformation, with legends shown on the right. (B) Species accumulation curves of Tibetan pig fecal samples, where error bars represent the range and shaded areas indicate the 95% confidence intervals. (C) Bubble plot of all viral species identified in this study, stratified into six categories across four farms. Bubble size denotes relative abundance, and bubble colors correspond to the four farms as indicated in the legend.

Supporting Information 3 Figure S2. Read mapping was performed as described in Figure 4D. For the remaining five strains (PLFe02_603, PLFe04_6559, etc.), views of their breakpoint insertion junction regions are shown for each. Multiple covering reads are present at each junction region for all strains.
