# Supplementary material for: Gut Virome of Tibetan Pigs Reveals the Diversity, Composition, and Distribution of Potential Novel Viruses/Variants
Source: Transbound Emerg Dis. 2025 Nov 19;2025:5191656. doi: 10.1155/tbed/5191656 (PMC12657093; doi:10.1155/tbed/5191656)
Supplement: Supporting Information 2 — Figure S1. (A) Rarefaction curves of species assignments generated in MEGAN v7.1.1 after log transformation, with legends shown on the right. (B) Species accumulation curves of Tibetan pig fecal samples, where error bars represent the range and shaded areas indicate the 95% confidence intervals. (C) Bubble plot of all viral species identified in this study, stratified into six categories across four farms. Bubble size denotes relative abundance, and bubble colors correspond to the four farms as indicated in the legend. [file 5191656.f2.pdf]

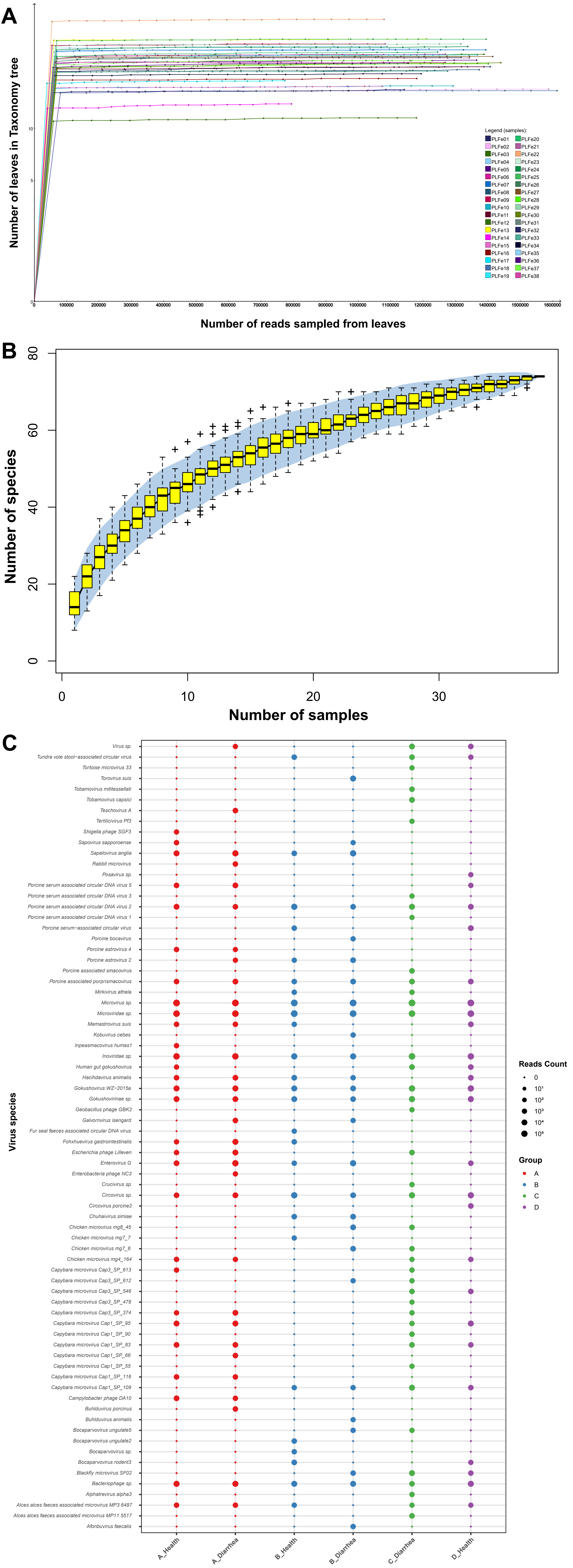

Supplementary Fig 1 (A) Rarefaction curves of species assignments generated in MEGAN v7.1.1 after log transformation, with legends shown on the right. (B) Species accumulation curves of Tibetan pig fecal samples, where error bars represent the range and shaded areas indicate the 95% confidence intervals. (C) Bubble plot of all viral species identified in this study, stratified into six categories across four farms. Bubble size denotes relative abundance, and bubble colors correspond to the four farms as indicated in the legend.
