# Supplementary material for: Gut Virome of Tibetan Pigs Reveals the Diversity, Composition, and Distribution of Potential Novel Viruses/Variants
Source: Transbound Emerg Dis. 2025 Nov 19;2025:5191656. doi: 10.1155/tbed/5191656 (PMC12657093; doi:10.1155/tbed/5191656)
Supplement: Supporting Information 3 — Figure S2. Read mapping was performed as described in Figure 4D. For the remaining five strains (PLFe02_603, PLFe04_6559, etc.), views of their breakpoint insertion junction regions are shown for each. Multiple covering reads are present at each junction region for all strains. [file 5191656.f3.pdf]

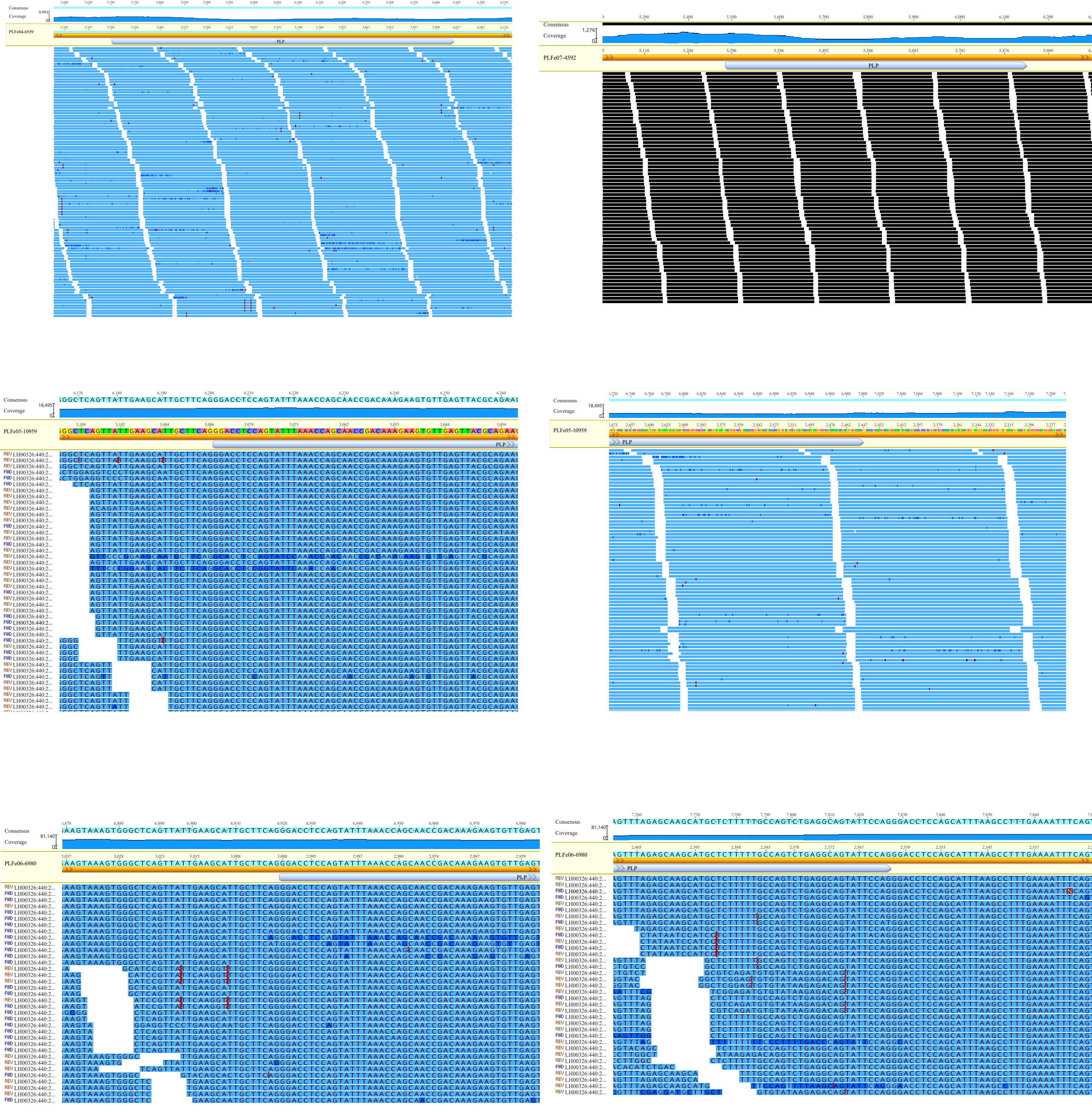

Supplementary Fig.2 Read mapping was performed as described in Fig. 4D. For the remaining five strains (PLFe02_603, PLFe04_6559, etc.), views of their breakpoint insertion junction regions are shown for each. Multiple covering reads are present at each junction region for all strains.
